# Supplementary material for: Ribonuclease 4 is associated with aggressiveness and progression of prostate cancer
Source: Commun Biol. 2022 Jun 25;5:625. doi: 10.1038/s42003-022-03597-1 (PMC9233706; doi:10.1038/s42003-022-03597-1)
Supplement: Supplementary file 2 — Supplementary Information [file 42003_2022_3597_MOESM2_ESM.pdf]

# Supplementary Figure 1

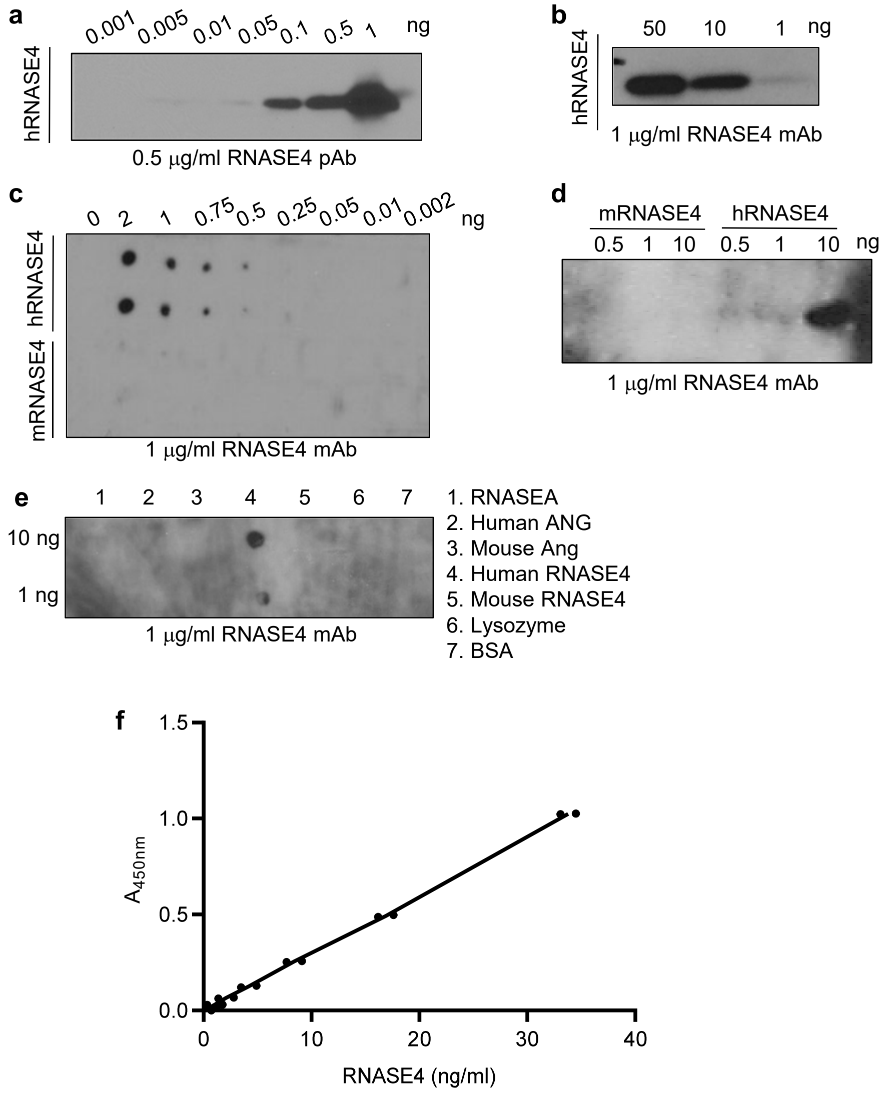


**Supplementary Fig.1. Sensitivity and specificity of RNASE4 polyclonal antibodies (pAb) and mAb. a,** Detection of human RNASE4 protein by RNASE4 pAb in immunobloting. At 0.5 μg/ml, RNASE4 pAb was able to detect as low as 50 pg RNASE4. **b,** Detection of human RNASE4 protein by RNASE4 mAb in immunoblotting. At 1 μg/ml, RNASE4 mAb was able to detect as low as 1 ng RNASE4. **c,** Detection of human and mouse RNASE4 protein by RNASE4 mAb in dot blotting. At 1 μg/ml, RNASE4 mAb was able to detect 0.5 ng human RNASE4 but not 2 ng mouse RNASE4 in dot blotting. **d,** Detection of human and mouse RNASE4 protein by RNASE4 mAb in immunoblotting. At 1 μg/ml, RNASE4 mAb was able to detect 1 ng human RNASE4 but not 10 ng mouse RNASE4 in immunoblotting. **e,** Detection of RNASEA, human and mouse ANG, human and mouse RNASE4, lysozyme, and BSA by RNASE4 mAb in dot blotting. At 1 μg/ml, RNASE4 mAb was able to detect 1 and 10 ng human RNASE4, but not anything else. **f,** A typical standard curve of RNASE4 sandwich ELISA using the above RNASE4 pAb and mAb.

# Supplementary Figure 2

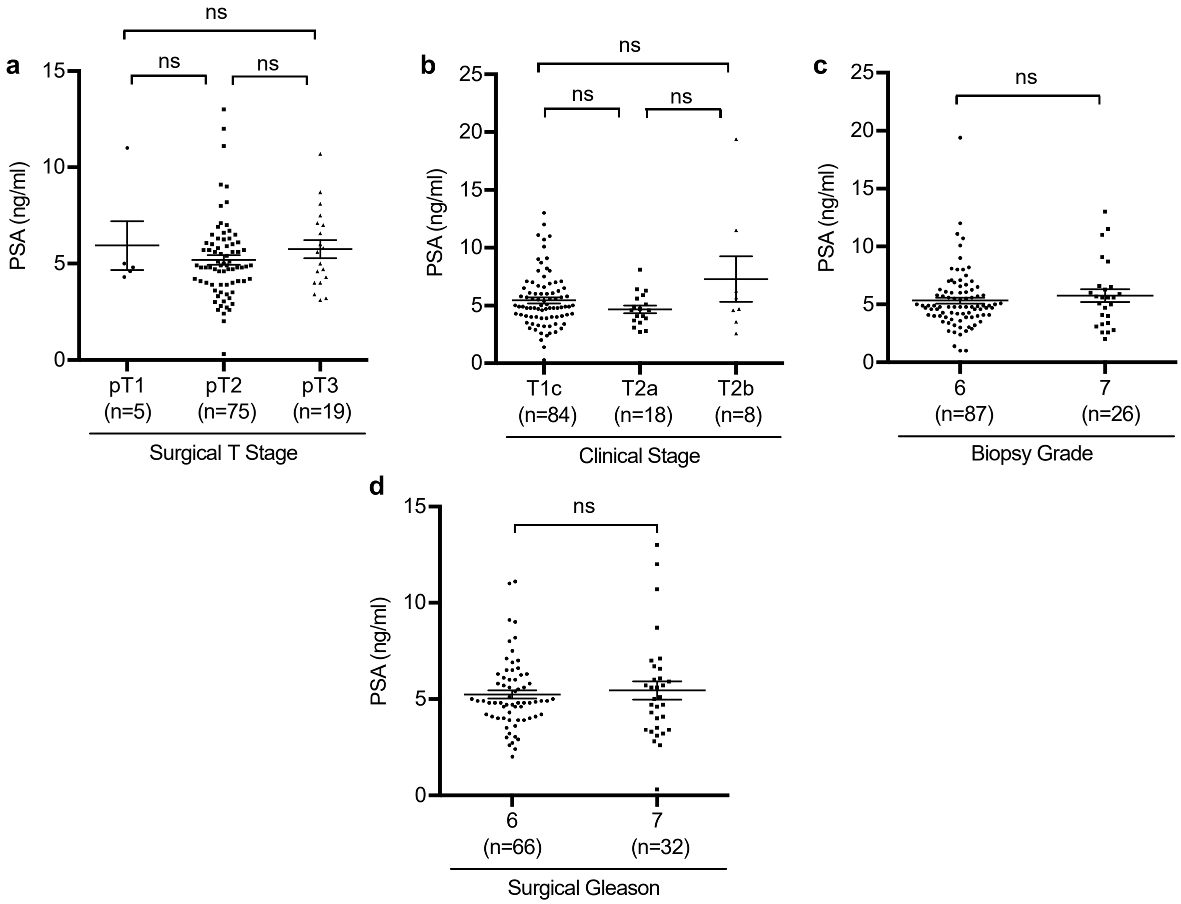


**Supplementary Fig. 2. PSA level is not correlated with poor prognosis.** Lack of correlation of plasma PSA level with tumor surgical T-stage (**a**), clinical stage (**b**), biopsy grade (**c**), and surgical Gleason (**d**). No significant differences were found by one-way ANOVA (**a,b**) and two-tailed Student's *t* test (**c,d**) analyses.

# Supplementary Figure 3

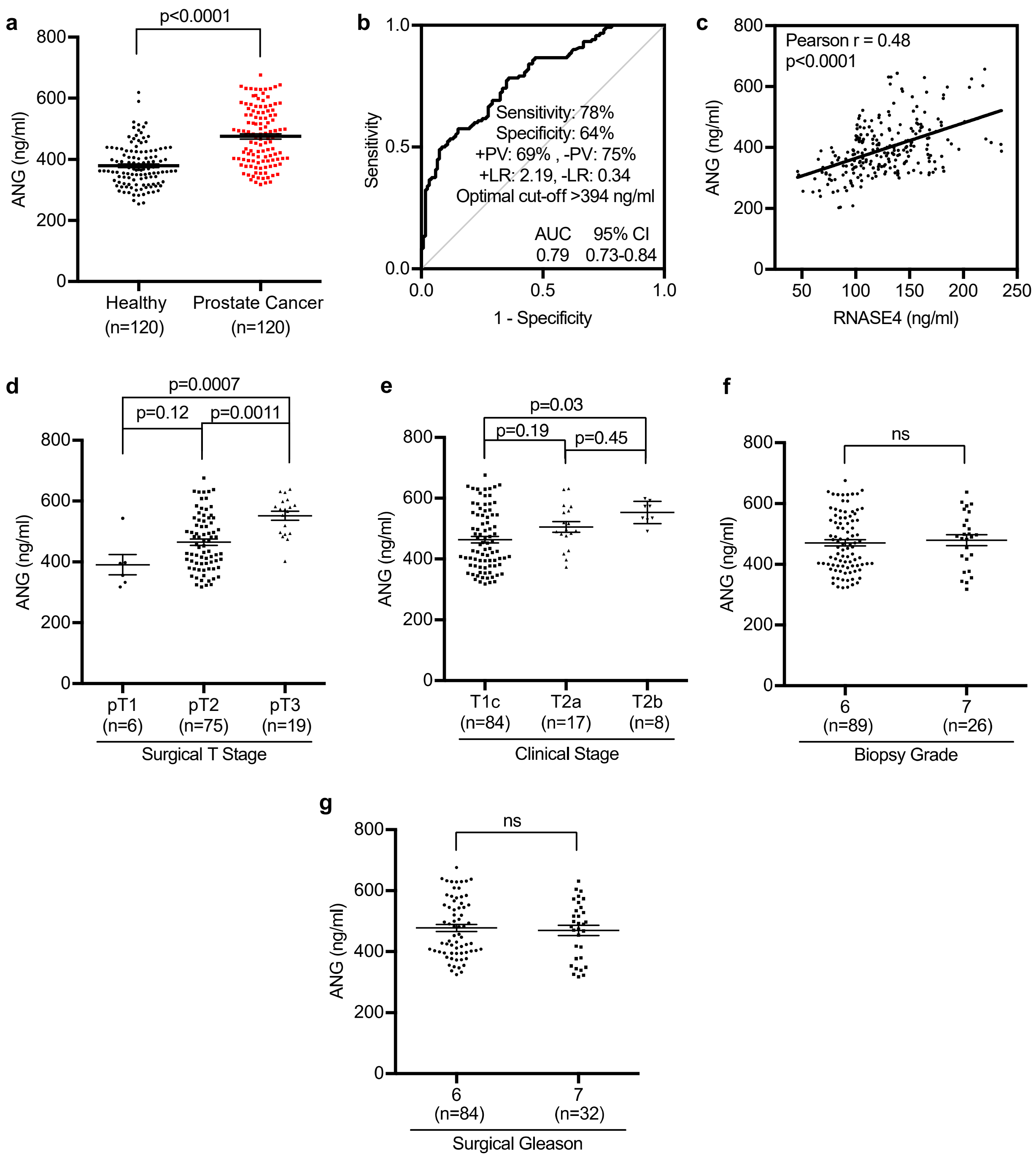


**Supplementary Fig. 3. ANG level is elevated in the plasma of prostate cancer patients and is correlated only with surgical T stage. a,** Levels of ANG protein in the plasma healthy control subjects (n=120) and prostate cancer patients (n=120). ANG amounts were determined by ELISA. Each dot represents an individual sample. Lines mark the median values and interquartile ranges. **b,** Operating Characteristic Curve (ROC) analysis. AUC, area under the curve; +PV, positive predictive value; –PV, negative predictive value; +LR, positive likelihood ratio; -LR, negative likelihood ratio. **c,** Correlation between RNASE4 and ANG in all plasma samples (n=240). (**d-g**) Plasma ANG levels in prostate cancer patients stratified by surgical T stage (**d**) clinical stage (**e**) biopsy grade (**f**), and surgical Gleason (**g**). Statistically significant differences were found only between pT1 and pT3, pT2 and pT3 (**d**), and between T1c and T2b (**e**). Statistical analyses used were one-way ANOVA (**d,e**) and two-tailed Student's *t* test (**a,f,** and **g**). ns= not significant.

# Supplementary Figure 4

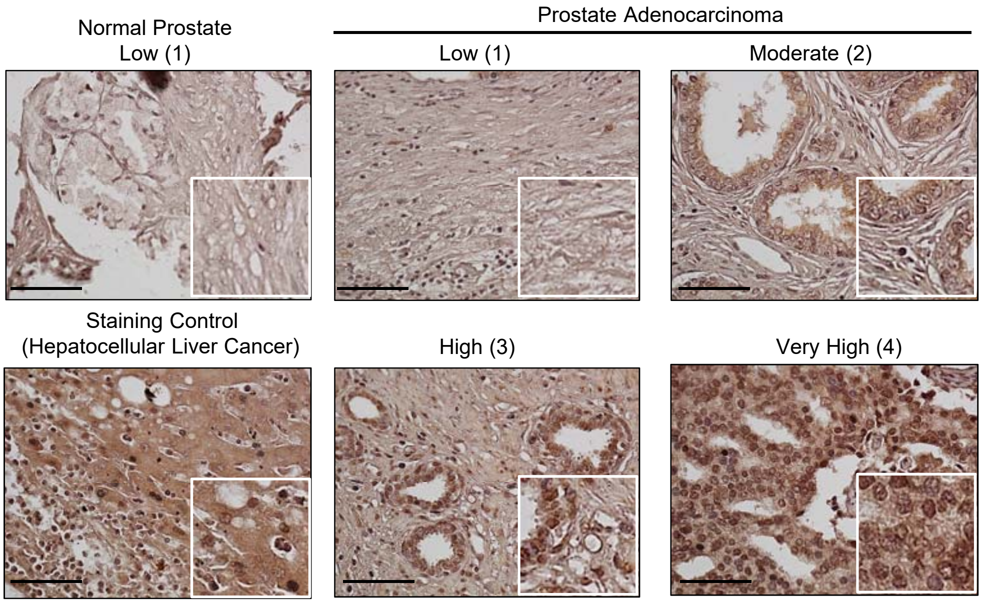


**Supplementary Fig. 4. RNASE4 IHC scoring in tissue micro array.** A semi-quantitative scoring scale was used to score staining density of RNASE4 in the tissue microarray. IHC score is indicated in parenthesis. Staining was ranked from 1 (low) to 4 (very high). RNASE4 staining was low in normal prostate tissue. Hepatocellular liver cancer tissue was used as positive staining control. Scale bars = 50 μm.

Supplementary Figure 5

**a** RNASE4 Expression in Bittner Multi-cancer Dataset

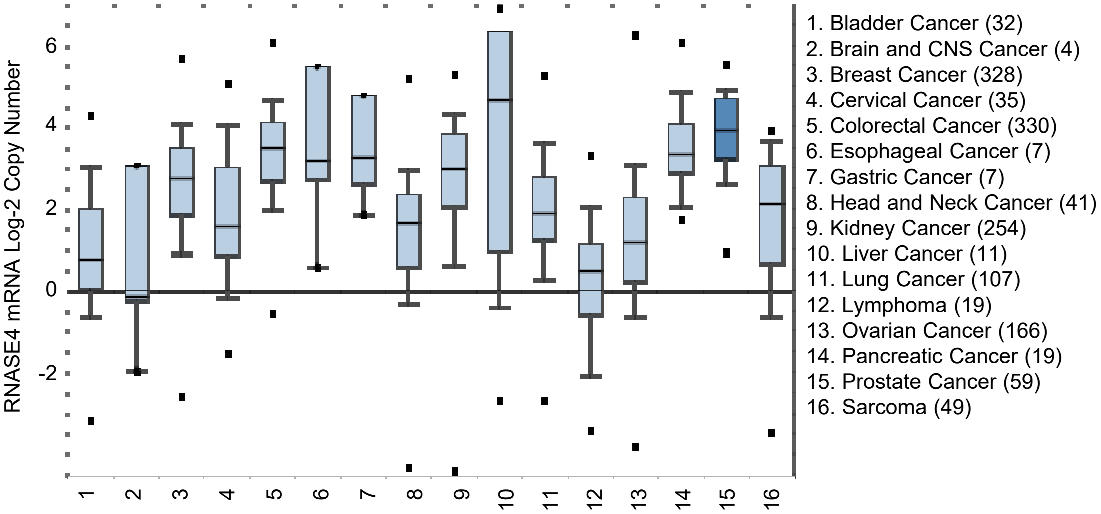


**b** RNASE4 Expression in Su Multi-cancer Dataset

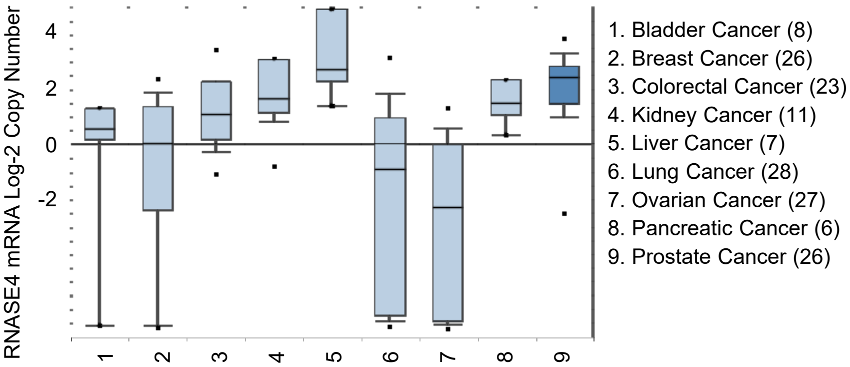


**c** **Prostate Cancer**

| Multi-cancer Dataset | Fold change | *p*-value | n |
|---|---|---|---|
| Bittner | 3.88 | 5.58E-17 | 59 |
| Su | 2.40 | 1.70E-11 | 26 |

**Supplementary Fig. 5. Bioinformatics analyses of *RNASE4* expression level in human cancers. a,** *RNASE4* mRNA level in different human cancers from Bittner Multi-cancer dataset. **b,** *RNASE4* mRNA level in different human cancers from Su multi-cancer dataset. The boxes represent the medians (black middle line) limited by the 25th (Q1) and 75th (Q3) percentiles. The whiskers are the upper and lower adjacent values, which are the most extreme values within Q3+1.5(Q3−Q1) and Q1−1.5(Q3−Q1), respectively. The number of patient samples is indicated in parenthesis. **c,** *p*-values and fold changes of *RNASE4* overexpression in prostate cancer from the two datasets.

Supplementary Figure 6

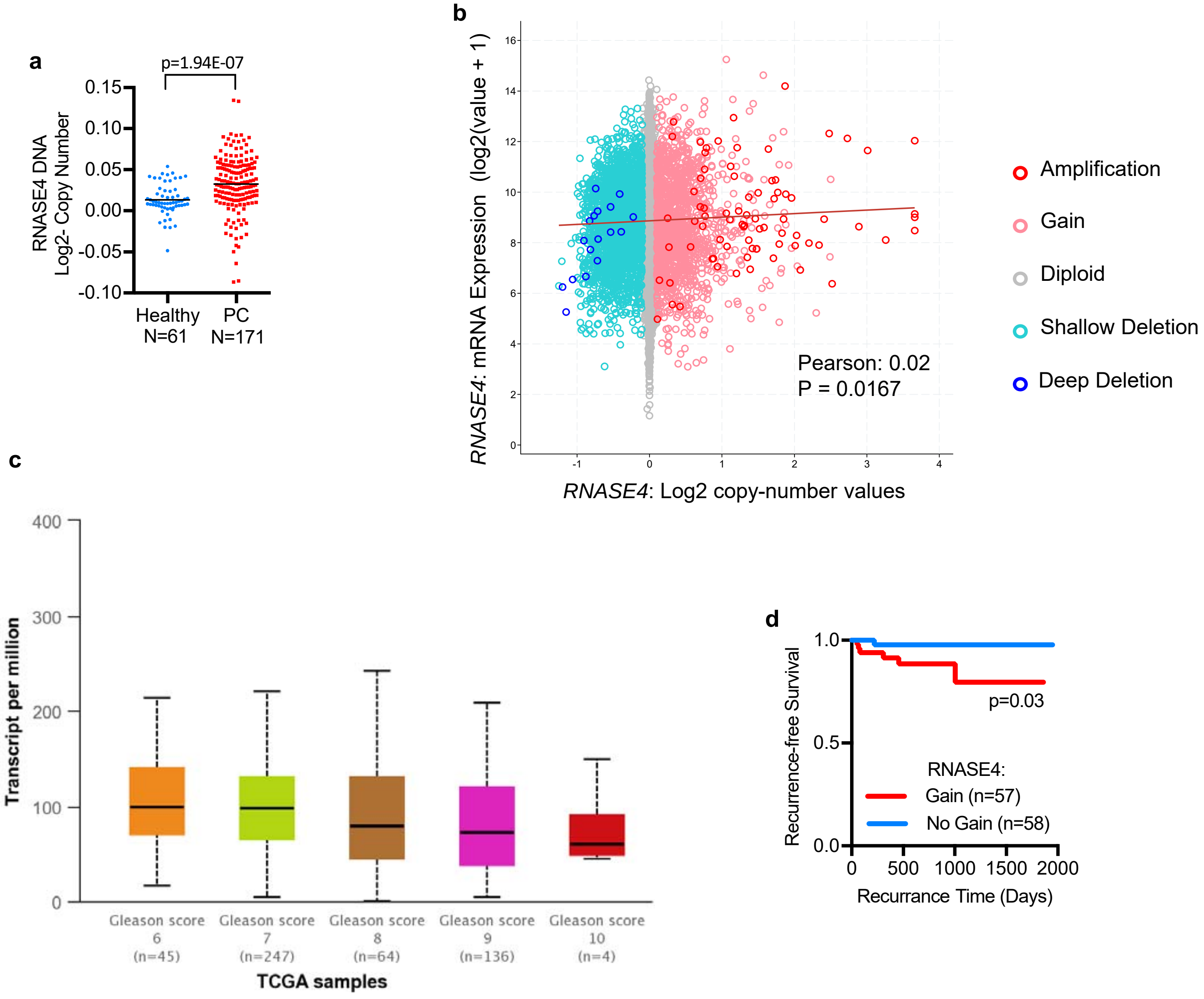


**Supplementary Fig. 6. Gain of *RNASE4* and *AXL gene copy numbers* is associated with poor outcome of prostate cancer. a,** Bioinformatics data of *RNASE4* DNA copy number variations from TCGA database (Oncomine.org) among healthy control subjects and prostate cancer (PC) patients. **b,** Correlation between *RNASE4* DNA copy number and *RNASE4* mRNA level analyzed by cBioPortal from TCGA dataset. **c,** *RNASE4* transcript levels in prostate cancers of different stage from TCGA database. **d,** Kaplan–Meier survival curves show the reverse correlation of the gain in gene copy number of *RNASE4* with recurrence-free prostate cancer patient survival. Two-tailed Student's *t* test and log-rank (Mantel-Cox) test were used for statistical analysis.

# Supplementary Figure 7

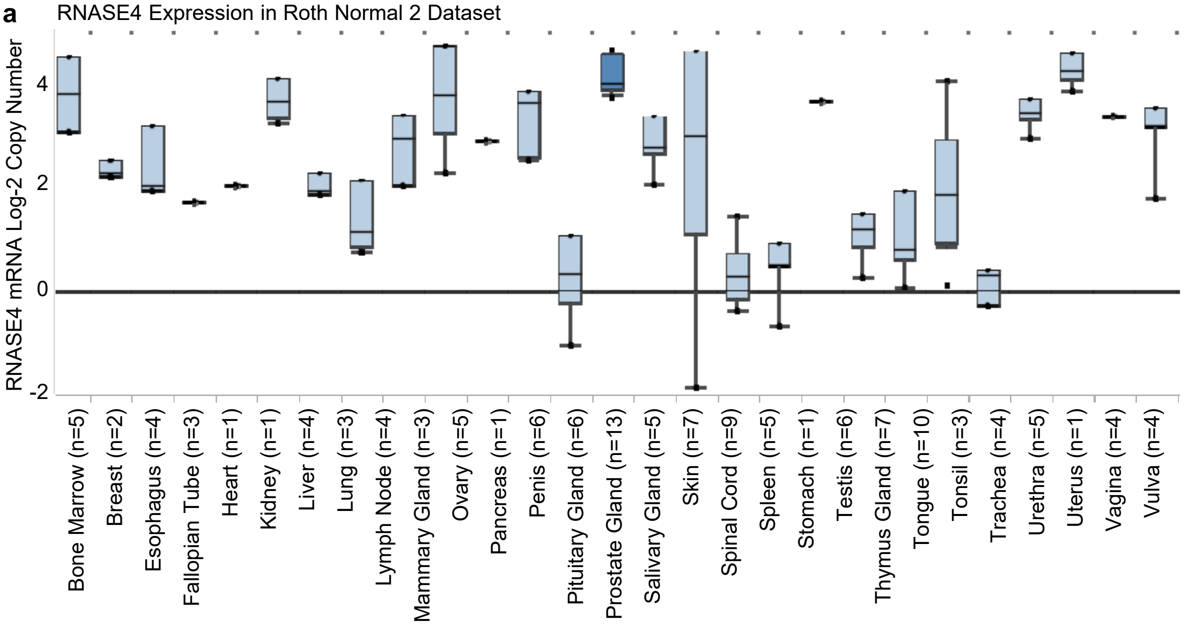


**b**

| **Organ Type** | **Fold Change** | ***p*-Value** | **n** |
|---|---|---|---|
| Prostate Gland | 4.33 | 6.21E-23 | 13 |
| Urethra | 4.39 | 4.81E-10 | 5 |
| Trachea | 2.34 | 1.54E-4 | 4 |
| Liver | 2.84 | 5.24E-4 | 4 |
| Ovary | 2.82 | 0.012 | 5 |
| Penis | 2.28 | 0.012 | 6 |
| Salivary Gland | 1.57 | 0.023 | 5 |

**Supplementary Fig. 7. Bioinformatics analyses of *RNASE4* mRNA level in normal human organs. a,** Organs with higher *RNASE4* mRNA level than the average of all human organs. The boxes represent the medians (black middle lines) limited by the 25th (Q1) and 75th (Q3) percentiles. The whiskers are the upper and lower adjacent values, which are the most extreme values within Q3+1.5(Q3−Q1) and Q1−1.5(Q3−Q1), respectively. The number of patient samples is indicated in parenthesis. The horizontal line at 0 marks average *RNASE4* mRNA level of all organs. *RNASE4* mRNA level in the prostate gland is 4.33 fold higher compared to other healthy organs ($p=6.21 \times 10^{-23}$). **b,** *p*-values and fold changes of *RNASE4* mRNA in the 7 healthy human organs with the highest expression.

# Supplementary Figure 8

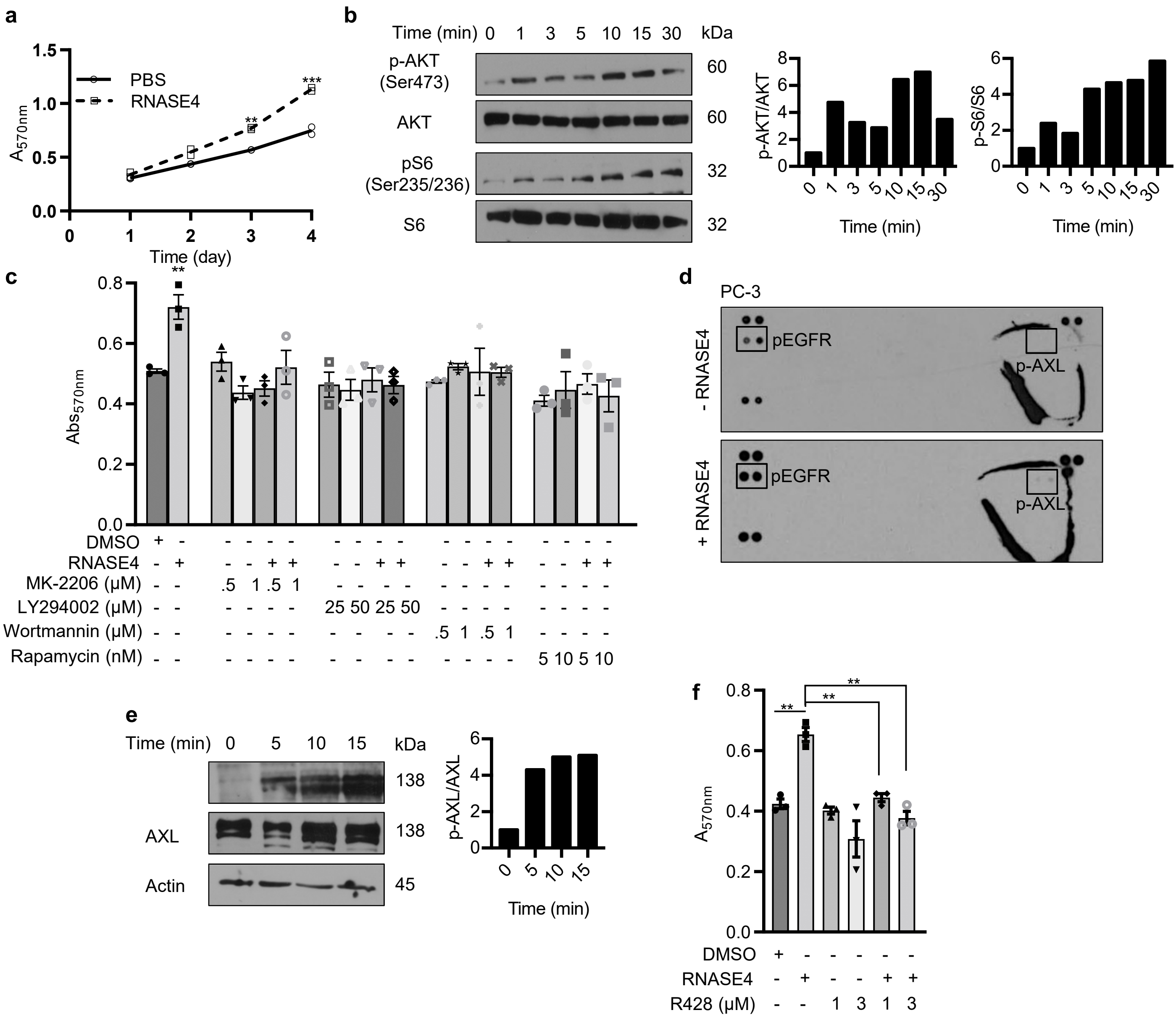


**Supplementary Fig. 8. RNASE4 induces cell proliferation and phosphorylation of AKT and S6. a,** Exogenous RNASE4 (1μg/ml) stimulates PC-3 cells proliferation in the presence of 2% FBS. Cell numbers were determined by MTT assay. **b,** Time course of AKT and S6 phosphorylation of serum-starved PC-3 cells by RNASE4 (1 μg/ml). Left panels, immunoblots; right panels, quantification of p-AKT and p-S6 normalized to total AKT and S6 by Image J. **c,** Effect of AKT inhibitor MK-2206, PI3K inhibitors LY294002 and Wortmannin, and mTOR inhibitor Rapamycin on RNASE4-induced PC-3 cell proliferation. Cells were pre-incubated with the inhibitors at the indicated concentrations for 1 hour prior to be stimulated by RNASE4 (1 μg/ml) for 3 days. Cell numbers were determined by MTT assay. **d,** Human phospho-RTK antibody array analysis of starved PC-3 cells treated with or without 1 μg/ml RNASE4 for 5 minutes. **e,** Time course of AXL phosphorylation stimulated by RNASE4 in PC-3 cells. Serum-starved PC-3cells were treated with 1 μg/ml RNASE4 for different time. Cell lysates were analyzed for total AXL and phopho-AXL by immunoblotting. Left panels, immunoblots; right panel, quantification phospho-AXL normalized to total AXL by Image J analysis. **f,** Effect of AXL inhibitor R428 on RNASE4-induced PC-3 cell proliferation. Serum-starved PC-3 cells were incubated with 1 or 3 μM R428 for 3 hours and then stimulated by 1 μg/ml of RNASE4 for 3 days. Cell numbers were determined by MTT assay. Data shown are means ± SEM of a representative experiments in triplicates of 3 independent repeats. ***p* ≤ 0.01 and ****p* ≤ 0.001, by unpaired two-tailed Student's *t* test.

# Supplementary Figure 9

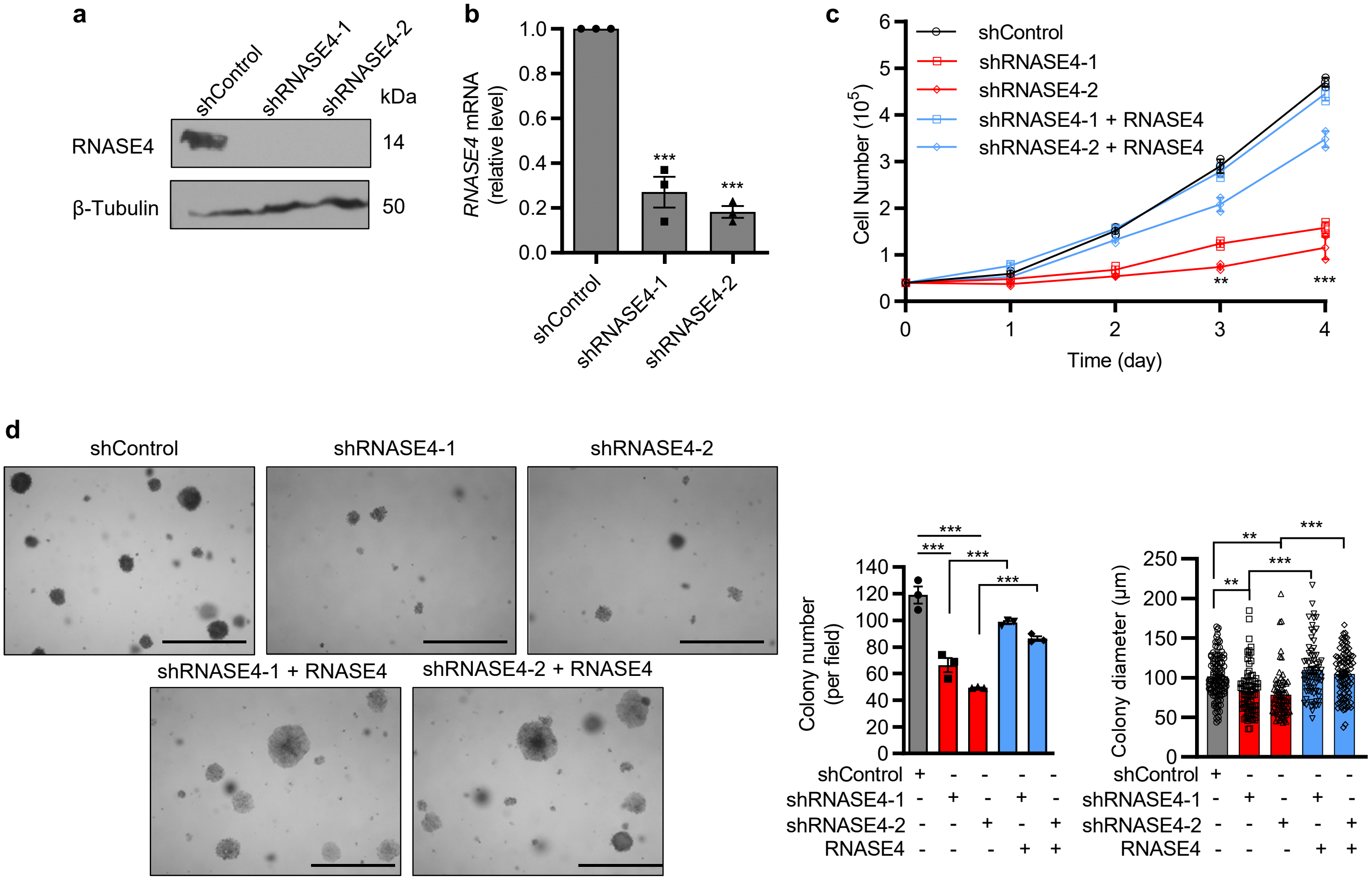


**Supplementary Fig. 9. *RNASE4* knockdown inhibits DU145 cell proliferation *in vitro* and colony formation in soft agar. a,** Immunoblot analysis of RNASE4 in DU145 cells stably transfected with shControl, shRNASE4-1, and shRNASE4-2. **b,** qRT-PCR analysis of *RNASE4* mRNA normalized to *GAPDH* in DU145 cells stably transfected with shControl, shRNASE4-1, and shRNASE4-2. **c,** Effect of *RNASE4* knockdown on cell proliferation. Cell numbers were counted by Coulter counter. Exogenouse RNASE4, when presented, was at the concentration of 1 µg/ml. Data shown are from a representative experiment in triplicates of 3 independent experiments. **d,** Effects of *RNASE4* knockdown on colonies formation and growth of DU145 cells in soft agar. The same numbers of control and *RNASE4* knockdown cells were seeded in soft agar, and grown in the absence or presence of 1 µg/ml of RNASE4 for 14 days. Colonies were counted and size measured on a Nikon Eclipse *ti* microscope. Top panels, representative images of colonies; scale bars =100 µm. Bottom panels, quantification of DU145 cell colonies and sizes in soft agar. Data shown are means ± SEM of five randomly selected fields per dish from a representative experiment. $^{**}p \leq 0.01$ and $^{***}p \leq 0.001$, by two-tailed Student's *t* test.

# Supplementary Figure 10

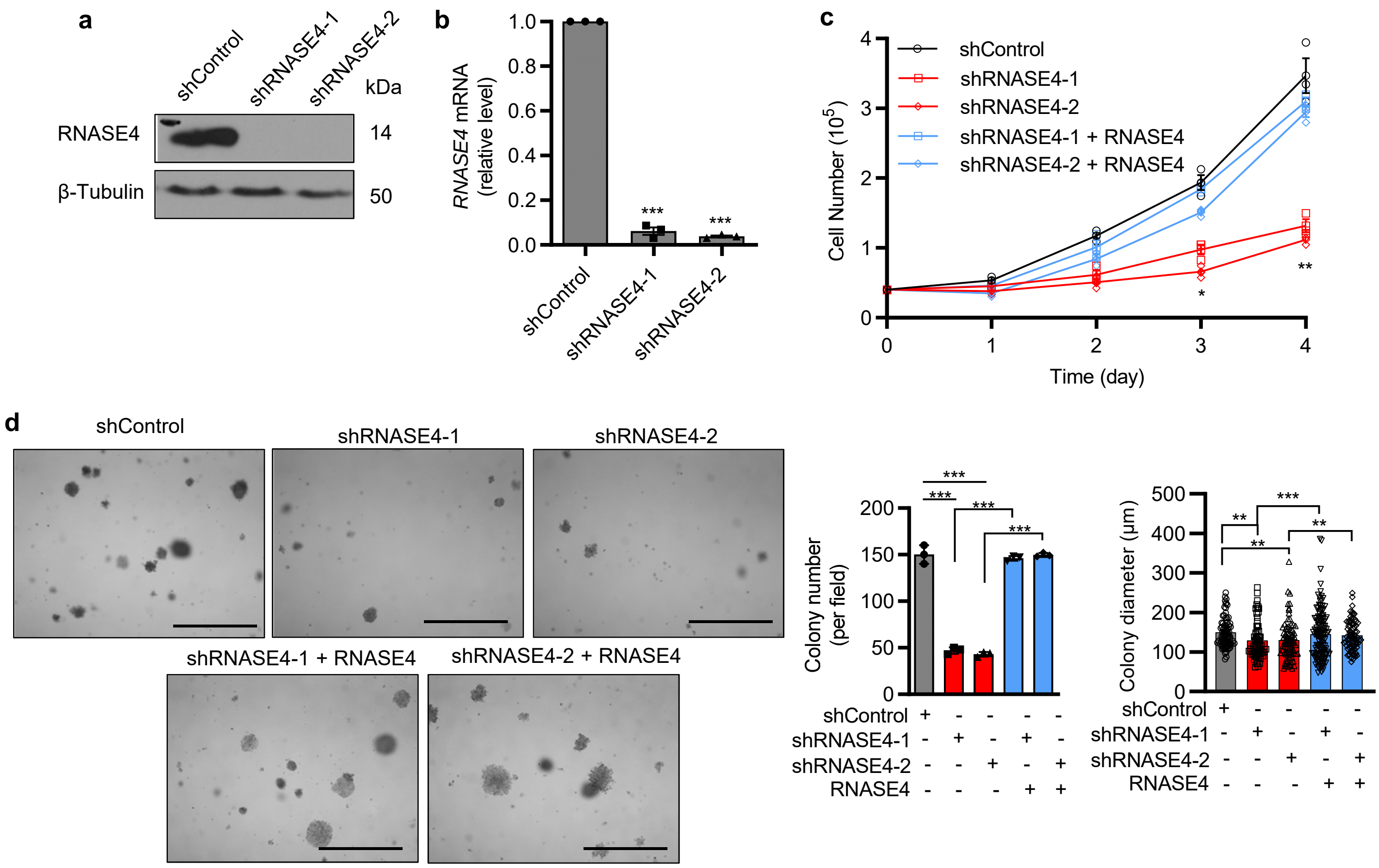


**Supplementary Fig. 10. *RNASE4* knockdown inhibits LNCaP cell proliferation *in vitro* and colony formation in soft agar. a,** Immunoblot analysis of RNASE4 in LNCaP cells stably transfected with shControl, shRNASE4-1, and shRNASE4-2. **b,** qRT-PCR analysis of *RNASE4* mRNA normalized to *GAPDH* in LNCaP cells stably transfected with shControl, shRNASE4-1, and shRNASE4-2. **c,** Effect of *RNASE4* knockdown on LNCaP cell proliferation. Cell numbers were counted by Coulter counter. Exogenouse RNASE4, when presented, was at the concentration of 1 µg/ml. Data shown are from a representative experiment in triplicates of 3 independent experiments. **d,** Effects of *RNASE4* knockdown on colonies formation and growth of LNCaP cells in soft agar. The same numbers of control and *RNASE4* knockdown cells were seeded in soft agar, and grown in the absence or presence of 1 µg/ml of RNASE4 for 14 days. Colonies were counted and size measured on a Nikon Eclipse *ti* microscope. Top panels, representative images of colonies; scale bars =100 µm. Bottom panels, quantification of LMCaP cell colonies and sizes in soft agar. Data shown are means ± SEM of five randomly selected fields per dish from a representative experiment. **$p \leq 0.01$ and ***$p \leq 0.001$, by two-tailed Student's *t* test.

Supplementary Figure 11

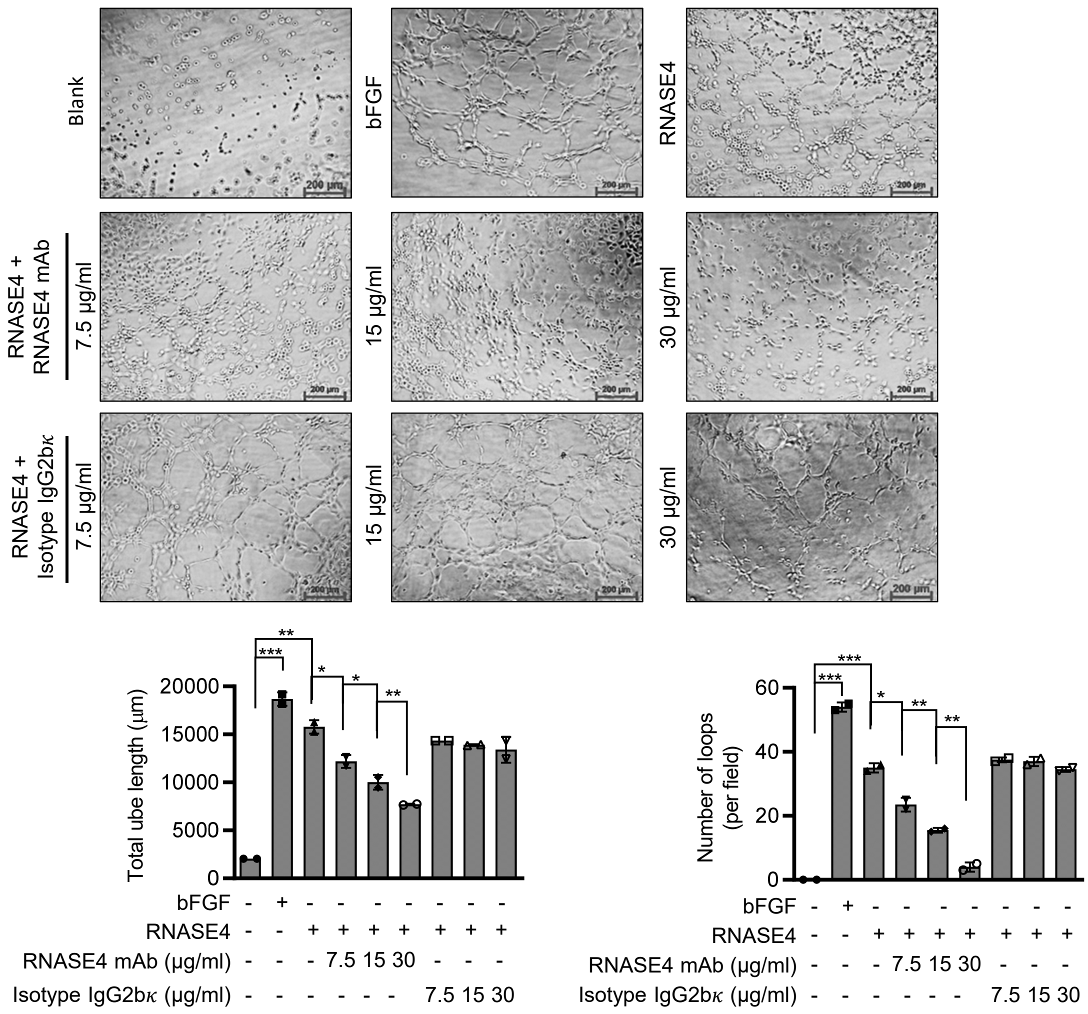


**Supplementary Fig. 11. RNASE4 mAb inhibits RNASE4-mediated angiogenesis in a dose-dependent manner.** HUVEC were cultured on Matrigel-coated wells in basal endothelial cell medium and stimulated with 1 μg/ml RNASE4 in the absence or presence of various concentrations of RNASE4 mAb or isotype control IgG2bκ for 4 hours. Untreated well was used as the negative control, and bFGF (5 ng/ml)-treated well was used as the postive control. Top panels, images of endothelial cell tubular structure of a representative experiment in duplicate of 3 independent repeats. Scale bar = 200 μm. Bottom panels, tube length and number of loops counted from 5 randomly selected areas. Data shown are means ± SEM. *$p \leq 0.05$, **$p \leq 0.01$, and ***$p \leq 0.001$, by two-tailed Student's *t* test.

# Supplementary Figure 12

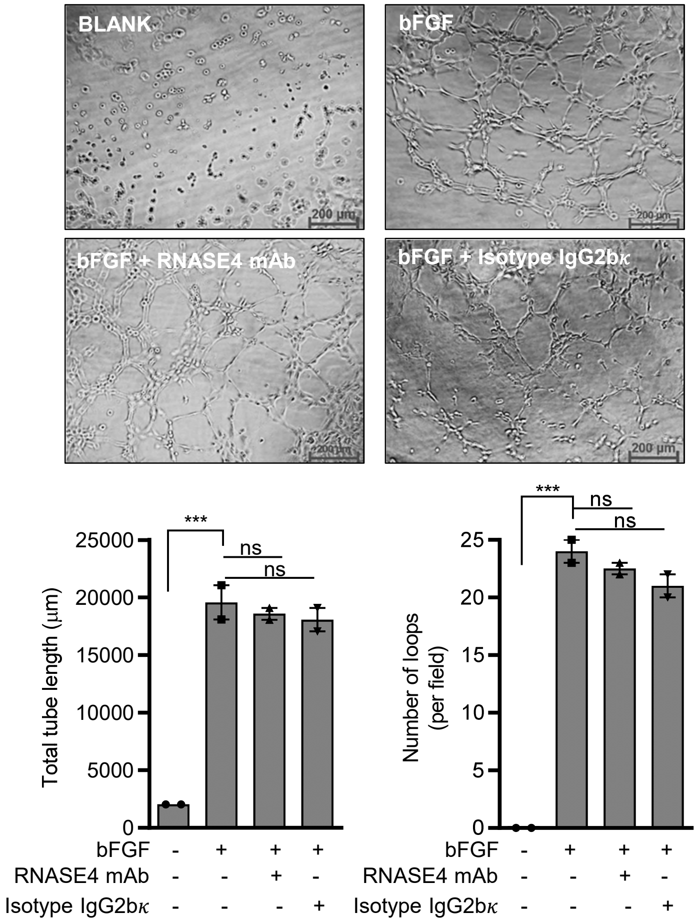


**Supplementary Fig. 12. RNASE4 mAb does not inhibit basic FGF (bFGF)-induced angiogenesis.** HUVEC were cultured on Matrigel-coated wells in basal endothelail cell culture medium and incubated in the absence or presence of 5 ng/ml of bFGF with 30 μg/ml of RNASE4 mAb or isotype control IgG2bκ for 4 hours. Untreated blank well was used as negative control. Top panels, images of endothelial cell tubular structure of a representative experiment in duplicate of 3 independent repeats. Scale bar = 200 μm. Bottom panels, tube length and number of loops counted from 5 randomly selected areas. Data shown are means ± SEM. ***$p \leq 0.001$ and ns = not significant, by two-tailed Student's *t* test.

Supplementary Figure 13

1g

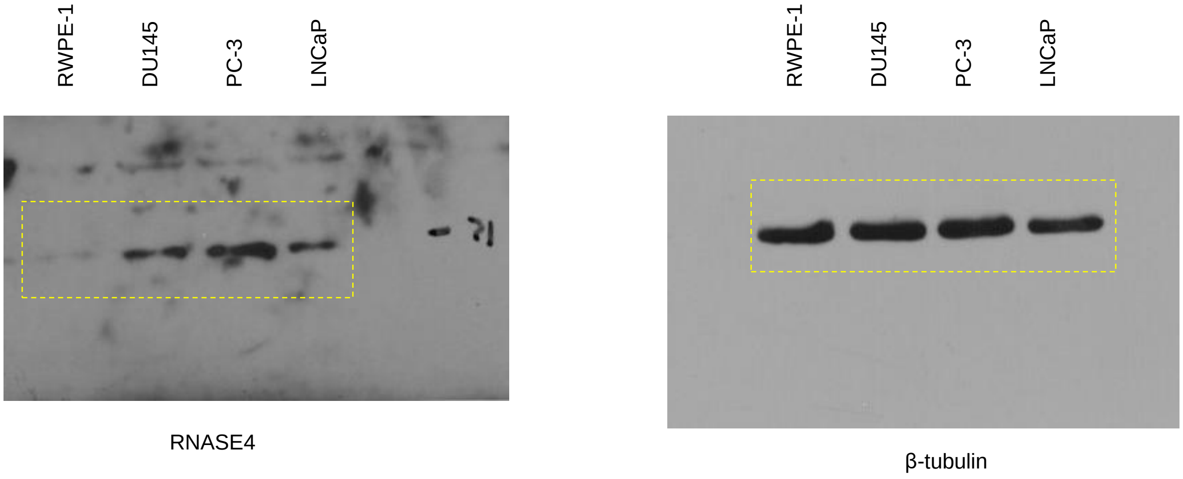


3c

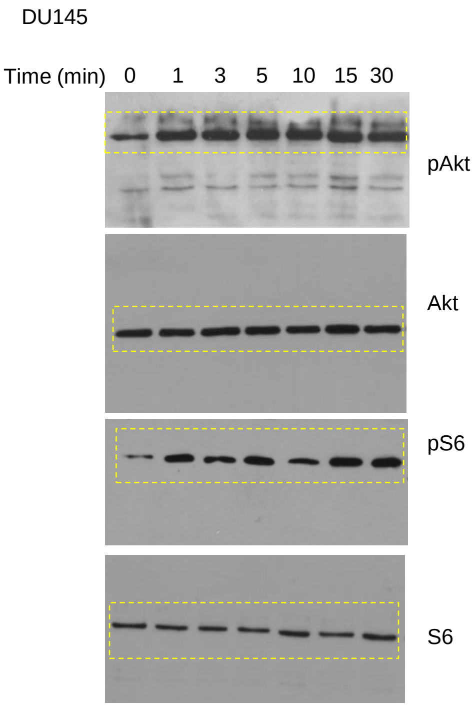


3d

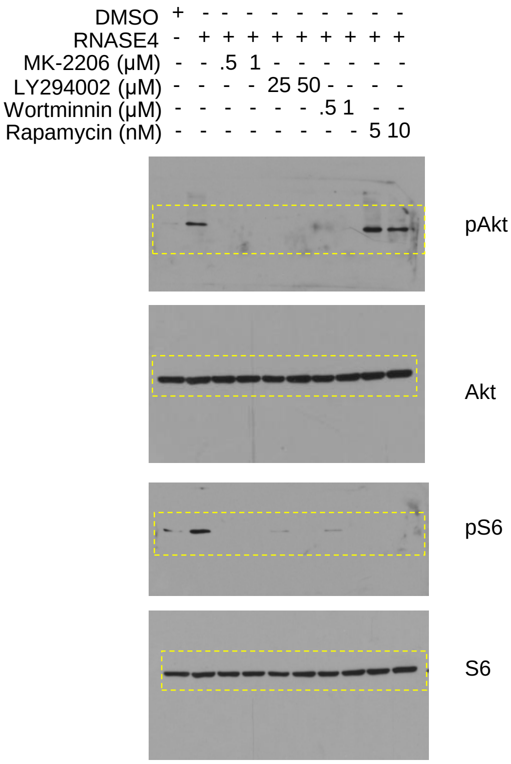


**Supplementary Fig. 13. Unedited gel for Fig. 1g, 3c, and 3d**

Supplementary Figure 14

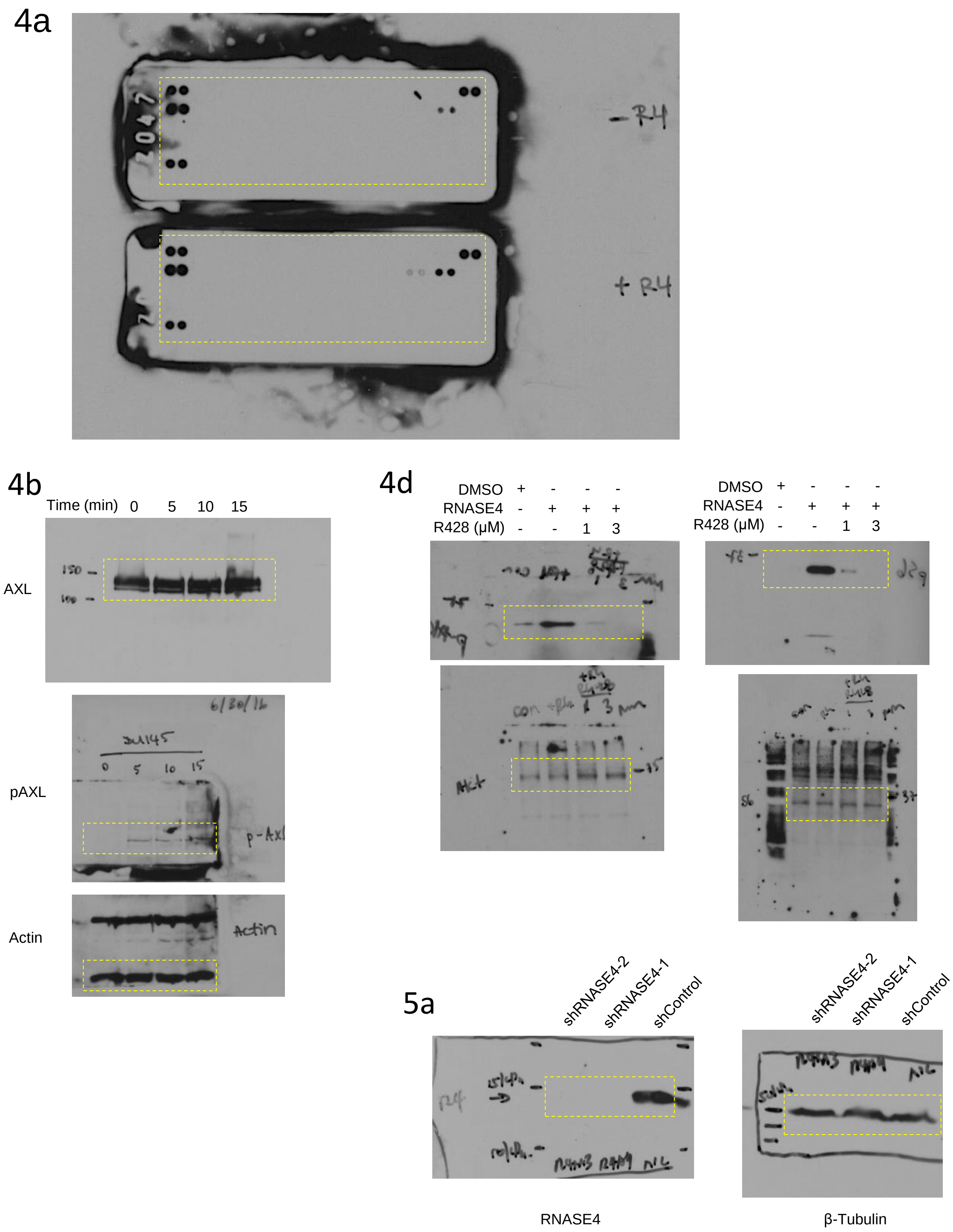


**Supplementary Fig. 14. Unedited gel for Fig. 4a, 4b, 4d, and 5a**

Supplementary Figure 15

S1a

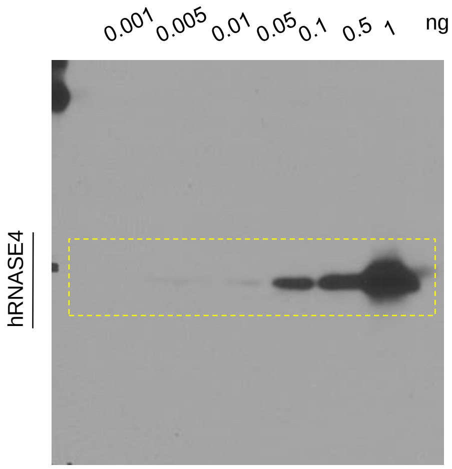


0.5 μg/ml RNASE4 pAb

S1b

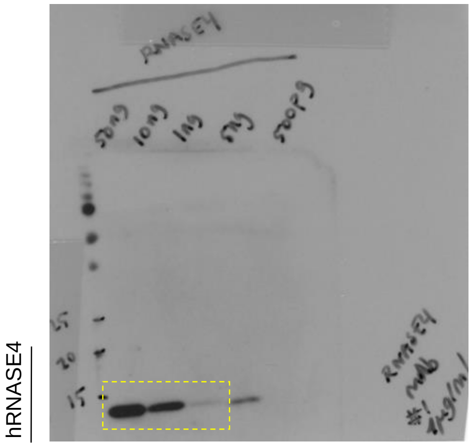


1 μg/ml RNASE4 mAb

S1c

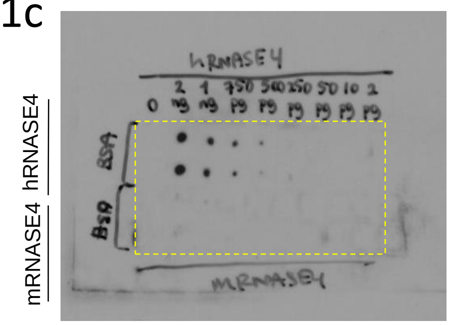


1 μg/ml RNASE4 mAb

S1d

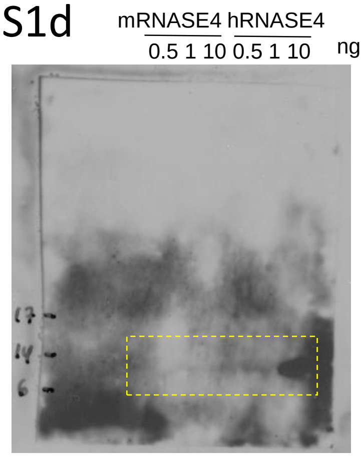


1 μg/ml RNASE4 mAb

S1e

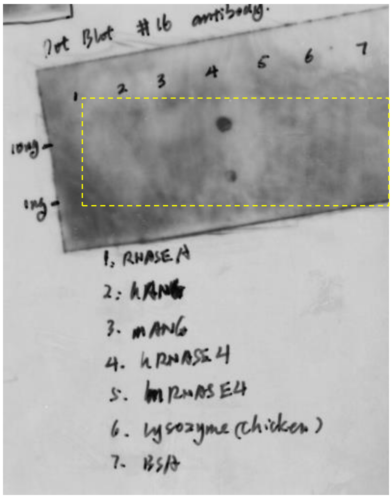

**Supplementary Fig. 15. Unedited gel for Supplementary Fig. 1**

Supplementary Figure 16

S8b

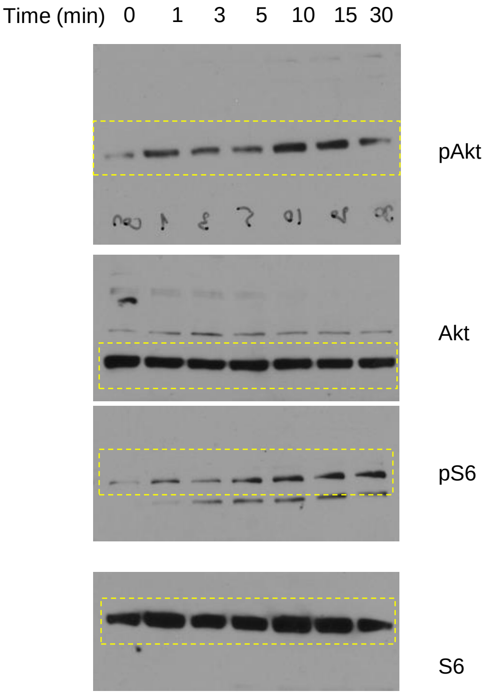


S8d

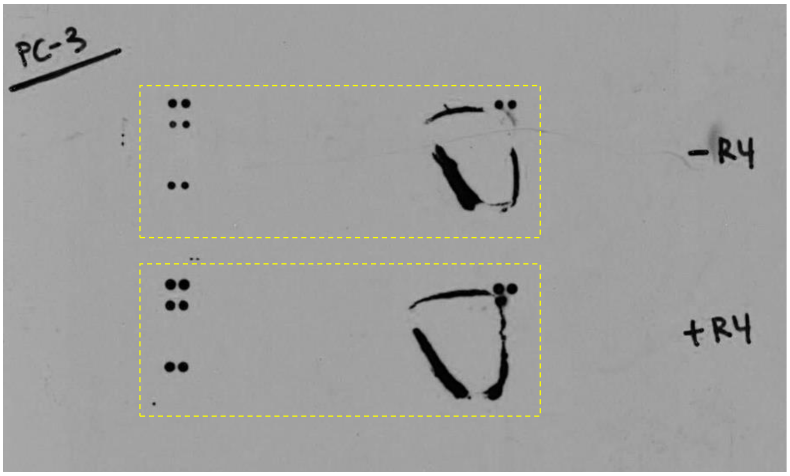


S9a

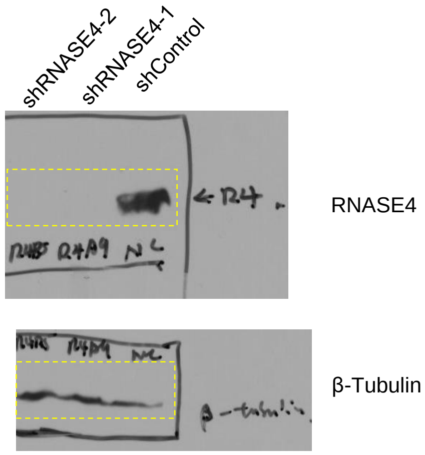


S8e

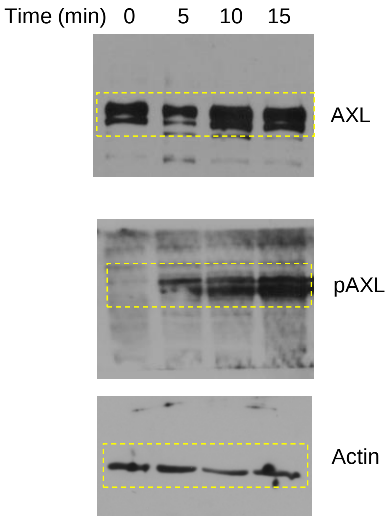


S10a

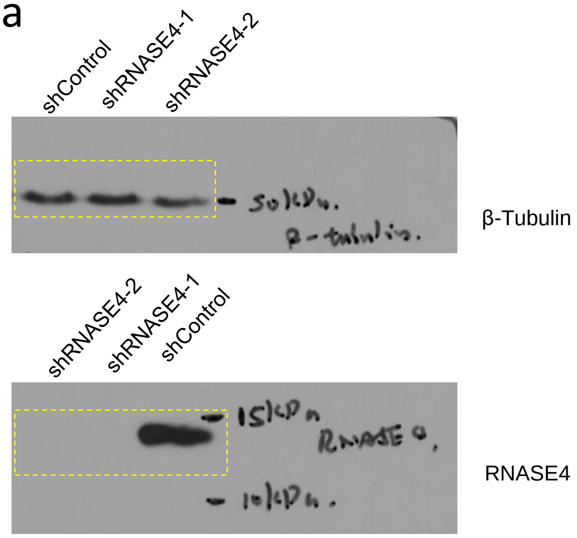


**Supplementary Fig. 16. Unedited gel for Supplementary Fig. 8a, 8d, 8e, 9a, and 10a**

**Supplementary Table 1. Demographic and clinical characteristics of study population**

| Characteristic | All subjects (N=240)[A] | Healthy (n=120)[A] | Prostate Cancer (n=120)[A] |
|---|---|---|---|
| **Age (years)** | | | |
| Mean | 58.6 | 57.4 | 59.8 |
| Min-Max | 42-86 | 42-86 | 43-77 |
| **Race** | | | |
| White/Non-Hispanic n (%) | 215 (86) | 104 (86.6) | 111 (92.5) |
| African-American n (%) | 16 (6.6) | 9 (7.5) | 7 (5.8) |
| Asian n (%) | 3 (1.2) | 2 (1.6) | 1 (0.8) |
| Other n (%) | 5 (2) | 4 (3.3) | 1 (0.8) |
| **PSA before biopsy (ng/ml)** | | | |
| Mean | 2.13 | 0.96 | 5.42 |
| Min-Max | 0.2-19.4 | 0.2-2.4 | 0.3-19.4 |
| ≤2 n (%) | 116 (48.3) | 111 (92.5) | 5 (4.2) |
| ≤4 n (%) | 146 (60.8) | 118 (98.3) | 28 (23.3) |
| <10 n (%) | 226 (94.2) | 118 (98.3) | 108 (90) |
| ≥10 n (%) | 8 (3.3) | 0 (0) | 8 (3.3) |

[A]Patient numbers may not add to the total sample size due to item nonresponse.

**Supplementary Table 2. Plasma RNASE4 level is not correlated with patient demographics**

| Clinical features | RNASE4 (ng/ml) Mean ± SEM | | | |
|---|---|---|---|---|
| **Age (years)** | **Healthy (n=120)[A]** | ***P*-value** | **Prostate Cancer (n=120)[A]** | ***P*-value** |
| 40-49 | 104.7 ± 4.1 (n=33) | | 144.4 ± 10.5 (n=6) | |
| 50-59 | 100.6 ± 2.9 (n=39) | p=0.41 | 154.7 ± 3.2 (n=62) | p=0.34 |
| 60-69 | 97.2 ± 3.9 (n=34) | p=0.19 | 153.2 ± 3.9 (n=40) | p=0.42 |
| 70-89 | 110.2 ± 4.7 (n=13) | p=0.46 | 137.2 ± 7.8 (n=12) | p=0.60 |
| **Race/Ethnicity** | | | | |
| White/Non-Hispanic | 102.3 ± 2.0 (n=104) | | 128.0 ± 2.3 (n=111) | |
| African-American | 94.4 ± 9.8 (n=9) | p=0.28 | 147.8 ± 9.3 (n=7) | p=0.13 |
| Asian | 95.0 ± 5.0 (n=2) | p=0.61 | 185.1 ± 0 (n=1) | n/a |
| Caribbean/West Indian/Hispanic | 84.7 ± 7.0 (n=2) | p=0.23 | 139.7 ± 0 (n=1) | n/a |
| Other | 132.9 ± 18.6 (n=2) | p=0.04 | n/a | n/a |

[A]Patient numbers may not add to the total sample size due to item nonresponse. n/a= information not available. Statistical analysis was performed by unpaired, two-tailed Student's *t* test.

**Supplementary Table 3. Assay validation of RNASE4 ELISA Precision[A]**

| | Intra-assay Precision | | | Inter-assay Precision | | |
|---|---|---|---|---|---|---|
| Sample | 1 | 2 | 3 | 1 | 2 | 3 |
| n | 12 | 12 | 12 | 12 | 12 | 12 |
| Mean (ng/ml) | 1.05 | 2.1 | 4.21 | 1.05 | 2.1 | 4.21 |
| Standard deviation | 0.013 | 0.008 | 0.011 | 0.016 | 0.035 | 0.041 |
| CV (%) | 4.9 | 1.8 | 2.3 | 5.9 | 9.3 | 8.5 |
| Precision (%) | 95.1 | 98.2 | 97.7 | 94.1 | 90.7 | 91.5 |

[A]Intra- and inter- assay precision of the established in-house sandwich RNASE4 ELISA. Three different RNASE4 protein concentrations were tested in 12 rows on one plate and in 12 different plates to determine intra-assay and inter-assay variability. Coefficients of variations (CV), demonstrating variability relative to the mean, were all less than 10% and within the allowable immunoassay range. CV: coefficient of variation.

**Supplementary Table 4. Assay validation of RNASE4 ELISA Recovery[A]**

| Sample type | Average % Recovery | Range |
|---|---|---|
| Plasma[B] (n=5) | 120 | 113-126 |

[A]Recovery was assessed by the ability to recover known amounts of recombinant RNASE4. The assay recovered average of 120% of the expected amounts of RNASE4 in plasma at working concentrations from 1 to 35 ng/ml.
[B]Plasma was diluted 1:10 prior to assay.

**Supplementary Table 5. Diagnostic accuracy of serum RNASE4 at various cut-offs**

| RNASE4 cut-off (ng/ml) | Sensitivity (%) | Specificity (%) | +PV (%) | -PV (%) | +LR | -LR | Accuracy (%) |
|---|---|---|---|---|---|---|---|
| 93 | 99 | 28 | 58 | 97 | 1.4 | 0.06 | 36 |
| 109 | 95 | 69 | 76 | 94 | 3.1 | 0.06 | 82 |
| 117 | 94 | 80 | 83 | 93 | 4.7 | 0.07 | 86 |
| 131 | 80 | 93 | 92 | 81 | 12 | 0.21 | 85 |
| 141 | 62 | 97 | 95 | 72 | 19 | 0.4 | 78 |

+PV: positive predictive value. -PV: negative predictive value. +LR: positive likelihood ratio. -LR: negative likelihood ratio.

**Supplementary Table 6. Relationship between plasma PSA amount and patient demographics**

| **Clinical features** | **PSA (ng/ml) Mean ± SEM** | | | |
|---|---|---|---|---|
| **Age (years)** | **Healthy (n=120)[A]** | ***P*-value** | **Prostate Cancer (n=120)[A]** | ***P*-value** |
| 40-49 | 0.7 ± 0.1 (n=33) | | 4.7 ± 0.7 (n=6) | |
| 50-59 | 1.0 ± 0.1 (n=39) | **p=0.02** | 5.9 ± 0.7 (n=62) | p=0.68 |
| 60-69 | 1.1 ± 0.1 (n=32) | **p=0.01** | 5.4 ± 0.4 (n=37) | p=0.67 |
| 70-89 | 1.1 ± 0.2 (n=13) | **p=0.02** | 6.7 ± 1.4 (n=12) | p=0.43 |
| **Race/Ethnicity** | | | | |
| White/Non-Hispanic | 1 ± 0.1 (n=102) | | 5.8 ± 0.5 (n=109) | |
| African-American | 0.8 ± 0.1 (n=9) | p=0.38 | 5.5 ± 0.7 (n=6) | p=0.86 |
| Asian | 1.8 ± 0.1 (n=2) | p=0.05 | 4.7 ± 0 (n=1) | n/a |
| Caribbean/West Indian/Hispanic | 0.8 ± 0.3 (n=2) | p=0.61 | 3.6 ± 0 (n=1) | n/a |
| Other | 0.8 ± 0.7 (n=2) | p=0.75 | n/a | n/a |

[A]Patient numbers may not add to the total sample size due to item nonresponse. n/a= information not available. Statistical analysis was performed by unpaired, two-tailed Student's *t* test. Significant *P*-values >0.05 were highlighted in bold.

**Supplementary Table 7. Relationship between plasma ANG amount and patient demographics**

| Clinical features | ANG (ng/ml) Mean ± SEM | | | |
|---|---|---|---|---|
| **Age (years)** | **Healthy (n=120)[A]** | ***P*-value** | **Prostate Cancer (n=120)[A]** | ***P*-value** |
| 40-49 | 390.2 ± 12.4 (n=33) | | 469.3 ± 32.6 (n=6) | |
| 50-59 | 397.4 ± 10.9 (n=39) | p=0.65 | 473.7 ± 12.1 (n=62) | p=0.91 |
| 60-69 | 356.3 ± 11.3 (n=32) | p=0.04 | 485.7 ± 15.8 (n=37) | p=0.69 |
| 70-89 | 357.0 ± 17.5 (n=13) | p=0.14 | 450.0 ± 25.7 (n=12) | p=0.66 |
| **Race/Ethnicity** | | | | |
| White/Non-Hispanic | 382.7 ± 7.0 (n=102) | | 473.8 ± 9.0 (n=109) | |
| African-American | 351.5 ± 14.9 (n=9) | p=0.20 | 481.6 ± 30.9 (n=6) | p=0.83 |
| Asian | 397.1 ± 28.6 (n=2) | p=0.78 | 598.7 ± 0 (n=1) | n/a |
| Caribbean/West Indian/Hispanic | 325.5 ± 33.1 (n=2) | p=0.26 | 447.4 ± 0 (n=1) | n/a |
| Other | 358.6 ± 3.7 (n=2) | p=0.63 | n/a | n/a |

[A]Patient numbers may not add to the total sample size due to item nonresponse. n/a= information not available. Statistical analysis was performed by unpaired, two-tailed Student's *t* test.

**Supplementary Table 8. Clinical characteristics of prostate cancer patients**

| Characteristic | Prostate Cancer (n=120)[A] | RNASE4 (ng/ml) |
|---|---|---|
| **Biopsy grade, n (%)** [B] | | |
| 6 | 89 (74.2) | 149.0 ± 2.8 |
| 7 | 26 (21.7) | 164.1 ± 3.9 |
| 8 | 2 (1.7) | 137.4 ± 19.6 |
| 9 | 2 (1.7) | 150.6 ± 13.6 |
| **Surgical Gleason, n (%)** [C] | | |
| 6 | 67 (55.8) | 147.8 ± 3.1 |
| 7 | 32 (26.7) | 157.8 ± 3.3 |
| 8 | 1 (0.8) | 205.7 |
| **Surgical T-stage, n (%)** [D] | | |
| pT1 (1c) | 6 (5) | 129.9 ± 3.6 |
| pT2 (2a-c) | 75 (62.5) | 149.5 ± 2.7 |
| pT3 (3a-b) | 19 (15.8) | 166.8 ± 5.6 |
| **Clinical T-stage, n (%)** [E] | | |
| T1c | 84 (70) | 148.0 ± 2.4 |
| T2a | 17 (14.1) | 166.6 ± 6.7 |
| T2b | 8 (6.7) | 178.5 ± 4.2 |

[A]Patient numbers may not add to the total sample size due to item nonresponse.
[B] Biopsy grade score 6 (well differentiated), 7 (moderately differentiated), 8-9 (poorly differentiated)
[C]Surgical Gleason score 6 (tumor somewhat resembles normal tissue), score 7-8 (tumor resembles normal tissue barely or not at all)
[D]Surgical T-stage pathalogic pT1 (tumor identified by a needle biopsy biopsy due to an elevated serum PSA), pT2 (tumor is confined to the prostate gland), pT3 (tumor extends through the prostate capsule)
[E]Tumor stage T1c (tumor identified by a needle biopsy due to an elevated serum PSA), T2a (tumor involves one-half of one lobe or less), T2b (tumor is in more than half of one lobe, but not both lobes)

**Supplementary Table 9. Histological characteristics of prostate cancer TMA cohort**

| Characteristic | Prostate Cancer (n=50)[A] |
|---|---|
| **Histological grade, n (%)[B]** | |
| 1-2 | 30 (60) |
| 3 | 20 (40) |
| **Tumor stage, n (%)[C]** | |
| T1-T2 | 27 (54) |
| T3-T4 | 23 (46) |
| **Gleason score n, (%)[D]** | |
| 2-3 | 27 (54) |
| 4-5 | 21 (42) |
| **Distant metastasis, n (%)[E]** | |
| M0 | 31 (62) |
| M1 | 15 (30) |
| **Lymph node metastasis, n (%)[F]** | |
| N0 | 31 (62) |
| N1-N2 | 17 (34) |

[A]Patient numbers may not add to the total sample size due to item nonresponse.
[B]Histological grade 1-2 (well-moderately differentiated), grade 3 (poorly differentiated)
[C]Tumor stage T1-T2 (tumor invades submucosa and muscularis propria), T3-T4 (tumor invades other organs or structures)
[D]Gleason score 2-3 (predominantly well-poorly formed glands) 4-5 (only poorly formed glands or lacking gland formation)
[E]Distant metastasis M0 (no distant metastasis), M1 (distant metastasis)
[F]Lymph node metastasis N0 (no regional lymph node metastasis) N1-N2 (metastasis in 1-4 or more lymph nodes).
