# Supplementary material for: Ribonuclease 4 is associated with aggressiveness and progression of prostate cancer
Source: Commun Biol. 2022 Jun 25;5:625. doi: 10.1038/s42003-022-03597-1 (PMC9233706; doi:10.1038/s42003-022-03597-1)
Supplement: Supplementary file 3 — Description of Additional Supplementary Files [file 42003_2022_3597_MOESM3_ESM.pdf]

Description of Additional Supplementary Files

Supplementary Data 1. Source data used for graphs and charts shown in main figures.

Supplementary Data 2. Source data used for graphs and charts shown in supplementary figures.
