# Supplementary material for: Ribonuclease 4 is associated with aggressiveness and progression of prostate cancer
Source: Commun Biol. 2022 Jun 25;5:625. doi: 10.1038/s42003-022-03597-1 (PMC9233706; doi:10.1038/s42003-022-03597-1)
Supplement: Supplementary file 6 — Reporting Summary [file 42003_2022_3597_MOESM6_ESM.pdf]

nature portfolio

Corresponding author(s): Guo-fu Hu

Last updated by author(s): Jun 1, 2022

Reporting Summary

Nature Portfolio wishes to improve the reproducibility of the work that we publish. This form provides structure for consistency and transparency in reporting. For further information on Nature Portfolio policies, see our Editorial Policies and the Editorial Policy Checklist.

Statistics

For all statistical analyses, confirm that the following items are present in the figure legend, table legend, main text, or Methods section.

n/a	Confirmed	
☐	☒	The exact sample size (*n*) for each experimental group/condition, given as a discrete number and unit of measurement
☐	☒	A statement on whether measurements were taken from distinct samples or whether the same sample was measured repeatedly
☐	☒	The statistical test(s) used AND whether they are one- or two-sided<br>*Only common tests should be described solely by name; describe more complex techniques in the Methods section.*
☒	☐	A description of all covariates tested
☐	☒	A description of any assumptions or corrections, such as tests of normality and adjustment for multiple comparisons
☐	☒	A full description of the statistical parameters including central tendency (e.g. means) or other basic estimates (e.g. regression coefficient) AND variation (e.g. standard deviation) or associated estimates of uncertainty (e.g. confidence intervals)
☐	☒	For null hypothesis testing, the test statistic (e.g. *F*, *t*, *r*) with confidence intervals, effect sizes, degrees of freedom and *P* value noted<br>*Give P values as exact values whenever suitable.*
☒	☐	For Bayesian analysis, information on the choice of priors and Markov chain Monte Carlo settings
☒	☐	For hierarchical and complex designs, identification of the appropriate level for tests and full reporting of outcomes
☒	☐	Estimates of effect sizes (e.g. Cohen's *d*, Pearson's *r*), indicating how they were calculated

Our web collection on statistics for biologists contains articles on many of the points above.

Software and code

Policy information about availability of computer code

Data collection	No commercial or custom code was used to collect data in this study
Data analysis	An in silico analysis on RNASE4 mRNA expression of Bittner Multi-cancer (n = 1,911), Su Multi-cancer (n = 174) and Roth Normal 2 (n = 504) datasets in a public cancer microarray database Oncomine (http://www.oncomine.org) was performed. The relationship between RNASE4 copy number amplification and mRNA expression from TCGA dataset was analyzed by cBioPortal for Cancer Genomics website (http://cbioportal.org). Comparisons between 2 groups were analyzed by 2-tailed Student's t test and comparisons of multiple groups were done by 1-way ANOVA, post hoc intergroup comparisons, and Tukey's test. Kaplan-Meier survival curves were analyzed using log-rank test. Receiver operating characteristic (ROC) curves were generated by MedCalc and Prism. Regression analyses were performed by MedCalc and Stata Software.

For manuscripts utilizing custom algorithms or software that are central to the research but not yet described in published literature, software must be made available to editors and reviewers. We strongly encourage code deposition in a community repository (e.g. GitHub). See the Nature Portfolio guidelines for submitting code & software for further information.
